# Murine Bone Marrow Lin^−^Sca-1^+^CD45^−^ Very Small Embryonic-Like (VSEL) Cells Are Heterogeneous Population Lacking Oct-4A Expression

**DOI:** 10.1371/journal.pone.0063329

**Published:** 2013-05-17

**Authors:** Krzysztof Szade, Karolina Bukowska-Strakova, Witold Norbert Nowak, Agata Szade, Neli Kachamakova-Trojanowska, Monika Zukowska, Alicja Jozkowicz, Jozef Dulak

**Affiliations:** 1 Department of Medical Biotechnology, Faculty of Biochemistry, Biophysics and Biotechnology, Jagiellonian University, Krakow, Poland; 2 Jagiellonian Centre for Experimental Therapeutics, Jagiellonian University, Krakow, Poland; University of Minnesota Medical School, United States of America

## Abstract

Murine very small embryonic-like (VSEL) cells, defined by the Lin^−^Sca-1^+^CD45^−^ phenotype and small size, were described as pluripotent cells and proposed to be the most primitive hematopoietic precursors in adult bone marrow. Although their isolation and potential application rely entirely on flow cytometry, the immunophenotype of VSELs has not been extensively characterized. Our aim was to analyze the possible heterogeneity of Lin^−^Sca^+^CD45^−^ population and investigate the extent to which VSELs characteristics may overlap with that of hematopoietic stem cells (HSCs) or endothelial progenitor cells (EPCs). The study evidenced that murine Lin^−^Sca-1^+^CD45^−^ population was heterogeneous in terms of c-Kit and KDR expression. Accordingly, the c-Kit^+^KDR^−^, c-Kit^−^KDR^+^, and c-Kit^−^KDR^−^ subpopulations could be distinguished, while c-Kit^+^KDR^+^ events were very rare. The c-Kit^+^KDR^−^ subset contained almost solely small cells, meeting the size criterion of VSELs, in contrast to relatively bigger c-Kit^−^KDR^+^ cells. The c-Kit^−^KDR^−^FSC^low^ subset was highly enriched in Annexin V-positive, apoptotic cells, hence omitted from further analysis. Importantly, using qRT-PCR, we evidenced lack of Oct-4A and Oct-4B mRNA expression either in whole adult murine bone marrow or in the sorted of Lin^−^Sca-1^+^CD45^−^FSC^low^ population, even by single-cell qRT-PCR. We also found that the Lin^−^Sca-1^+^CD45^−^c-Kit^+^ subset did not exhibit hematopoietic potential in a single cell-derived colony *in vitro* assay, although it comprised the Sca-1^+^c-Kit^+^Lin^−^ (SKL) CD34^−^CD45^−^CD105^+^ cells, expressing particular HSC markers. Co-culture of Lin^−^Sca-1^+^CD45^−^FSC^low^ with OP9 cells did not induce hematopoietic potential. Further investigation revealed that SKL CD45^−^CD105^+^ subset consisted of early apoptotic cells with fragmented chromatin, and could be contaminated with nuclei expelled from erythroblasts. Concluding, murine bone marrow Lin^−^Sca-1^+^CD45^−^FSC^low^ cells are heterogeneous population, which do not express the pluripotency marker Oct-4A. Despite expression of some hematopoietic markers by a Lin^−^Sca-1^+^CD45^−^c-Kit^+^KDR^−^ subset of VSELs, they do not display hematopoietic potential in a clonogenic assay and are enriched in early apoptotic cells.

## Introduction

Bone marrow (BM) contains populations of hematopoietic [1], [2] and non-hematopoietic stem cells [3]–[5]. It has been proposed several years ago that adult murine BM can be a source of homogenous population of rare pluripotent stem cells, named very small embryonic-like (VSEL) cells [6], [7], which could represent the most primitive BM cell subset [7], [8]. VSELs were characterized as small, Lin^−^Sca-1^+^CD45^−^ cells, expressing pluripotency markers, e.g. Oct-4. Then, cells of VSEL immunophenotype have also been detected by the same researchers in other adult organs both in mice [9] and humans [10].

There are several publications, by the same group, speculating that VSELs are crucial in tissue regeneration and longevity [11]–[14]. It was also suggested that VSELs can be differentiated toward the hematopoietic lineage [7]. However, hematopoietic potential of murine VSELs is still a matter of debate, as they can repopulate bone marrow only when expanded by a long-term *in vitro* culture on the feeder layer [7]. There is lack of evidence if the same could be repeated at single cell level to exclude false positive effects caused by cell sorting impurities. Furthermore, the hematopoietic potential of human cord-blood derived VSELs were recently undermined [15].

The immunophenotype of murine VSELs is poorly characterized, as it overlaps to some extent with that of hematopoietic stem cells (HSC) (Lin^−^Sca-1^+^) or endothelial progenitor cells (EPC) (CD45^−^Sca-1^+^). On the other hand, features such as small size and CD45 negativity can also be shared with nuclei expelled from erythroblasts during erythropoiesis [16], [17]. Because murine VSEL characteristics include only one positive marker, extended identification is needed to select more homogenous population with better stemness properties. Moreover, some technical concerns should be taken into consideration when detecting Oct-4A expression, as Oct-4 pseudogenes are spread over the genome, which may impede actual Oct-4A mRNA detection in adult organisms [18].

The aim of our study was to assess possible heterogeneity of murine VSELs in expression of certain HSC and EPC surface markers, verify the expression of Oct-4A, and check their hematopoietic potential at the single cell level.

## Materials and Methods

### Ethics Statement

All animal procedures and experiments were performed in accordance with national and European legislations, after approval by the First Local Ethical Committee on Animal Testing at the Jagiellonian University in Krakow (approval number: 56/2009).

### BM Isolation and Staining

Bone marrow cells were isolated by flushing the tibias and femurs from 3–5 months old C57Bl/6×FVB mice, with RPMI-1640 medium (Lonza) containing 10% fetal bovine serum (FBS) (Lonza). The cell suspensions were filtered with 70 µm strainer, depleted of erythrocytes by use of a hypotonic solution, centrifuged (600 *g*, 10 minutes, 4°C), resuspended in PBS (Lonza) with 2% FBS, and stained for 20 minutes on ice. We tested also the isolation method with additional step of collagenase digestion, as described by Morikawa and coworkers [19]. We did not find, however, an improvement in efficacy of isolation of subpopulations of interest (data not shown).

Following antibodies were used for cell phenotyping: CD34-FITC (clone RAM34, BD Biosciences), Sca-1-PE-Cy7 (clone D7, BD Biosciences), Sca-1-PE-Cy5 (clone D7, Biolegend), c-Kit-APC-eFluor780 (clone 2B8, eBioscience, USA), KDR-FITC or KDR-APC (clone Avas12alpha1, BD Biosciences), CD45-V450, CD45-FITC, CD45-APC, CD45-APC-Cy7 (clone 30F-11, BD Biosciences), CD105-PE-Cy7 (clone MJ7/18, Biolegend), CD150-APC (clone TC15-12F12.2, Biolegend), CD48-PerCP-Cy5.5 (clone HM-48-1, Biolegend), CD49f-APC (clone GoH3, Biolegend), CD90.2-APC (clone 30-H12, Biolegend), CD71-FITC (clone RI7217, Biolegend), Annexin V-FITC (cat. 4830-01-K, Trevigen). For detection of lineage-determined cells the following set of antibodies was applied: CD45R-PE (clone RA3-6B2, BD Biosciences), Ly6G and Ly6C-PE (clone RB6-8C5, BD Biosciences), TCRγδ-PE (clone GL3, BD Biosciences), TCRβ-PE (clone H57-597, BD Biosciences), CD11b-PE (clone M1/70, BD Biosciences), Ter119-PE (clone TER119, BD Biosciences). The stained cells were analyzed using LSRII flow cytometer (BD Biosciences), with FACSDiva (BD Biosciences) and FlowJo (TreeStar) software.

### ImageStream Analysis

The bone marrow cells were isolated and labeled as described above, with combination of CD45-FITC, Lin-PE, Sca-1-PE-Cy5, and c-Kit-APC-Cy7 antibodies, and analyzed by ImageStream X system (Amnis) with 40× and 60× objectives, using Inspire and Ideas software (Amnis). The single cells were gated by analyzing Aspect Ratio Intensity vs. Area parameters, calculated on the brightfield channel. The cells that were in focus were selected by analyzing the Gradient RMS parameter calculated on brightfield channel. Further gating strategy was done in a similar way to flow cytometric analyses (Fig. S1). The diameter of the cells (Fig. S2) was calculated using Ideas 4.0 software, by given formula: 2×(Area/π)^0.5^.

### Single Cell Hematopoietic Colony Forming *in vitro* Assay

BM-derived cells were isolated, stained as described above using combination of CD45-FITC, Lin-PE, Sca-1-PE-Cy5, CD105-PE-Cy7 or c-Kit-APC-Cy7 antibodies, and sorted with MoFlo XPD (Beckman Coulter) cell sorter. Only DAPI-negative cells with integral membrane were chosen, using gating strategy presented in Fig. S3. Cells were sorted to non-adherent round-bottom 96-well plates (Greiner Bio-One), with a single cell per well, into 150 µl of serum-free expansion medium (StemSpan® SFEM, Stem Cell Technologies), supplemented with 20% of BIT 9500 Serum Substitute (Stem Cell Technologies), 0.1% of Ex-Cyte supplement (Millipore), and cytokine mix: murine stem cell factor (mSCF, Peprotech), human thrombopoietin (hTPO, Peprotech), murine interleukin-3 (mIL-3, Peprotech), and human erythropoietin (hEPO, Sigma-Aldrich), all at the concentration of 20 ng/ml. Cells were cultured for 10 days (37°C, 5% CO_2_), then wells with colonies were counted, the colonies were harvested, diluted with PBS to final volume of 250 µl, cytospined (1,000 rpm, 10 minutes, room temperature), air-dried, and stained using Wright’s method with Hemacolor Kit (Merck).

### Hematopoietic Differentiation with OP9 Co-culture

OP9 cells (2,500 per well of 96-well plate) were seeded in αMEM medium supplemented with 20% FBS (Lonza), sodium bicarbonate (2.2 g/L, Sigma), β-mercaptoethanol (55 µM), penicillin (100 U/ml, Sigma) and streptomycin (100 µg/ml streptomycin, Sigma). After 3 days, when they reached confluence, medium was changed and Lin^−^Sca-1^+^CD45^−^FSC^low^ or SKL CD45^+^ populations were directly sorted over the OP9 feeder layer.

First strategy implied sorting Lin^−^Sca-1^+^CD45^−^FSC^low^ or SKL CD45^+^ cells from the BM of C57BL/6-Tg(UBC-GFP)30Scha/J mice ubiquitously expressing green fluorescent protein (GFP) reporter transgene (1,000 cells per well with confluent OP9 layer). After 4 days of co-culture, medium and trypsinized cells were collected, washed with PBS containing 2% FBS, and the GFP^+^ cells were sorted on non-adherent round-bottom 96-well plates, with a single cell per well, into 150 µl of serum-free expansion medium, supplemented with 20% of BIT 9500 serum substitute, mSCF, mIL-3, hEPO and hTPO as described above. After 14 days wells with GFP^+^ colonies were counted.

In the second strategy, we sorted aliquots of 1, 25, 50, 75 and 100 Lin^−^Sca-1^+^CD45^−^FSC^low^ or SKL CD45^+^ cells per well with confluent OP9 layer. After 5 days of co-culture, medium and trypsinized cells from a well were washed, centrifuged, suspended in 150 µl of serum-free expansion medium, supplemented with 20% of BIT 9500 serum substitute, mSCF, mIL-3, hEPO and hTPO as described above and seeded in well on non-adherent round-bottom 96-well plates. After 14 days wells with colonies were counted. Frequency of cells with clonogenic potential within studied population was estimated using the limiting dilution method. Log_10_ of percentage of negative wells in given sorted cell concentration was plotted against sorted cell concentration. Next, by using linear regression and Poisson distribution statistics, the number of cells in a given population consisting of one clonogenic cell was calculated as that corresponding to value of 37% negative wells.

### TUNEL Assay on Sorted Cells

Cells were sorted on poli-L-lysine coated slides according to protocol proposed by Ema and co-workers [20]. Chromatin fragmentation was examined with TUNEL assay (FragEL™ DNA Fragmentation Detection Kit, Calbiochem) according to the manufacturer’s instruction. The cells were analyzed using Nikon Eclipse Ti (Nikon) fluorescence microscope.

### Erythroblast *in-vitro* Enucleation

Process of erythroblast enucleation was studied *ex vivo* following the method described by Yoshida and co-workers [16]. Bone marrow cells were isolated and stained using CD45-FITC and Ter119-PE antibodies. The 225,000 of CD45^−^Ter119^+^ erythroblasts were sorted per well of 24-well plate by means of MoFlo XPD cell sorter. Part of sorted population was stained immediately after sort (with Lineage, CD45, Sca-1, c-Kit, CD105 antibodies, and Hoechst; CD45 and Ter119 antigens were labeled with the antibodies used for erythroblasts sorting) and analyzed by flow cytometry (LSR-II). Remaining sorted cells were incubated in αMEM medium (Lonza) with 10% FBS (37°C, 5% CO_2_). After 1 h, 2.5 h and 6 h cells were collected, stained as described above and analyzed by flow cytometry. Additionally, cells from the whole bone marrow were stained directly after isolation and analyzed in the same way.

### RNA Isolation, RT-PCR and Real–time PCR

Total cellular RNA was purified by phenol/chloroform extraction. Reverse transcription was performed with M-MuLV Reverse Transcriptase (Finnzymes) and oligo(dT) primers (Promega). When indicated, the DNase I treatment of RNA prior to reverse transcription was performed using the RNase-Free DNase Set (Qiagen) for 15 minutes in room temperature, followed by heat-inactivation.

PCR reaction was conducted with Taq polymerase (Promega) using the following conditions: 95°C for 5 minutes, 40 cycles of 95°C for 30 seconds, annealing temperature for 30 seconds, and 72°C for 45 seconds, with final elongation at 72°C for 5 minutes. Following primers were used in the study: Oct-4A Forward –5′-CCC CAA TGC CGT GAA GTT GGA GAA GGT-3′, Oct4B Forward –5′-ATG AAA GCC CTG CAG AAG GAG CTA GAA CA-3′, Oct4A and Oct4B Reverse –5′-TCT CTA GCC CAA GCT GAT TGG CGA TGT G-3′, according to Mizuno and Kosaka [21], EF-2 Forward –5′-GCG GTC AGC ACA CTG GCA TA-3′ Reverse –5′-GAC ATC ACC AAG GGT GTG CAG-3′ acted as endogenous control. Additionally, set of primers designed by Kucia et al. [6] was used: Oct-4 Forward –5′-ACC TTC AGG AGA TAT GCA AAT CG-3′, Oct-4 Reverse –5′-TTC TCA ATG CTA GTT CGC TTT CTC T-3′. Agarose gel electrophoresis (3% agarose in TAE buffer) of the PCR products was performed according to standard laboratory protocols.

Quantitative real-time PCR (qPCR) with melt curve analysis of the amplified products was carried out using the StepOne Plus cycler (Applied Biosystems) and SYBR® Green JumpStart™ Taq ReadyMix™ (Sigma-Aldrich).

Single cell qPCR was performed with AmpliSpeed system (Beckman Coulter Biomedical, Germany). The single cells per fields on AmpliGrid slides were sorted and dried overnight in 4 °C. Then, a reverse transcription reaction was carried out with NCode™ VILO™ miRNA cDNA Synthesis Kit (Life Technlogies, USA) directly on the slide on AmpliSpeed cycler. The obtained cDNA was used for qPCR reaction.

### Statistical Analysis

Data are presented as mean ± standard deviation of at least three independent experiments. Unpaired t-test was used to analyze differences when two groups were compared. One-way ANOVA with Bonferroni post-test was applied when more than two groups were compared. Significance of proportions was assessed using Fisher exact test. Results were considered as significant when p<0.05. The graphs design and statistical analysis were done using GraphPad Prism software (GraphPad Software).

## Results

### Subpopulations of Lin^−^Sca-1^+^CD45^−^ Cells in Murine BM

The murine VSELs, claimed to be pluripotent, were originally defined by the Lin^−^Sca-1^+^CD45^−^ phenotype and small size [6]. Here, we investigated if additional markers can be used to select more homogenous population with better stemness characteristics.

**Figure 1:**
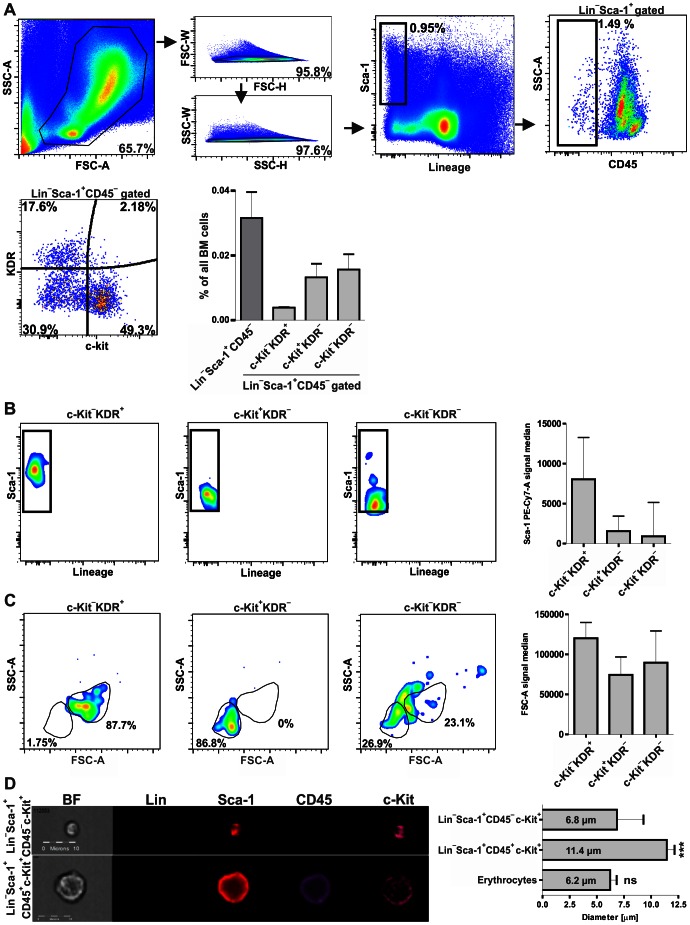
Heterogeneity of BM-derived Lin^−^Sca-1^+^CD45^−^ population. (**A**) c-Kit^−^KDR^+^, c-Kit^+^KDR^−^ and ^−^c-Kit^−^KDR^−^ subsets could be distinguished within Lin^−^Sca-1^+^CD45^−^ population. (**B**) c-Kit^−^KDR^+^ subpopulation showed higher Sca-1 expression comparing to c-Kit^+^KDR^−^ and c-Kit^−^KDR^−^ subpopulations. (**C**) c-Kit^+^KDR^−^ subset showed the most restricted FSC^low^ phenotype. c-Kit^−^KDR^−^ subpopulation also included FSC^low^ cells, while no such cells were visible in c-Kit^−^KDR^+^ subset. (**D**) ImageStream analysis of non-fixed cells revealed that diameter of the Lin^−^Sca-1^+^CD45^−^c-Kit^+^ cells is significantly lower than diameter of Lin^−^Sca-1^+^CD45^+^c-Kit^+^ cells and comparable to erythrocyte size. Each bar represents mean ± SD. *** - p<0.001 vs. Lin^−^Sca-1^+^CD45^−^c-Kit^+^.

Flow cytometry analysis revealed that within the Lin^−^Sca-1^+^CD45^−^ population, the c-Kit and KDR (Flk-1) surface expression can be used to recognize three distinct subsets: c-Kit^−^KDR^+^, c-Kit^+^KDR^−^ and c-Kit^−^KDR^−^ (Fig. 1A). A few c-Kit^+^KDR^+^ events were also detectable, but their number was too low to reliably confirm the presence of a distinct subpopulation. The frequency of total Lin^−^Sca-1^+^CD45^−^ cells in murine BM equaled 0.0316% ±0.0179% of all nucleated cells (Fig. 1A). Within Lin^−^Sca-1^+^CD45^−^ subsets, the c-Kit^−^KDR^+^ was the rarest subpopulation (0.0039% ±0.00014%), while c-Kit^+^KDR^−^ and c-Kit^−^KDR^−^ were more frequent (0.0132% ±0.0094% and 0.0156% ±0.0106%, respectively) (Fig. 1A).

Backgating of each Lin^−^Sca-1^+^CD45^−^ subset showed that c-Kit^−^KDR^+^ cells possessed higher Sca-1 expression comparing to c-Kit^+^KDR^−^ and c-Kit^−^KDR^−^ subpopulations (Fig. 1B). Importantly, according to FSC/SSC values, all cells characterized as c-Kit^−^KDR^+^ within Lin^−^Sca-1^+^CD45^−^ population were relatively big (Fig. 1C) and thus, could not be classified as VSELs. In contrast, entire population of c-Kit^+^KDR^−^ cells showed uniform low FSC/SSC values (Fig. 1C), whereas c-Kit^−^KDR^−^ cells did not exhibit homogenous FSC/SSC characteristics, being dispersed from very small to relatively big cells. The results indicated that within Lin^−^Sca-1^+^CD45^−^ subpopulation only the c-Kit positive fraction, and part of double negative subset, with its restricted FSC^low^SSC^low^ characteristic, fulfills size criterion of VSEL cells.

Next, the absolute size of the Lin^−^Sca-1^+^CD45^−^c-Kit^+^ was measured using the ImageStream system, as done previously for VSELs [22]. This method allowed gating of cells in similar manner to that presented for flow cytometric analysis (Fig. S1). The diameter of unfixed Lin^−^Sca-1^+^CD45^−^c-Kit^+^ was assessed as 6.8±2.4 µm and was significantly smaller (p<0.001) when compared to the diameter of hematopoietic progenitors (11.4±0.7 µm), defined as Lin^−^Sca-1^+^CD45^+^c-Kit^+^ (Fig. 1D). The diameter of red blood cells was 6.2±0.6 µm and did not differ significantly from Lin^−^Sca-1^+^CD45^−^c-Kit^+^ subpopulation (Fig. 1D). The size distribution of Lin^−^Sca-1^+^CD45^−^c-Kit^+^ clustered around the measured mean (44.2% of cells were included within 5–8 µm range), but events with diameter of 2–4 µm or bigger than 8 µm could be also detected (Fig. S2B). We found that the outliers were enriched in debris as visualized with Image Stream. Moreover, those with diameter between 2–4 µm were too small to confirm reliably their cellular morphology by Image Stream examination (Fig. S2B).

Additionally, the size of Lin^−^Sca-1^+^CD45^−^c-Kit^−^ subpopulation was assessed. Consistent with flow cytometry analysis (Fig. 1C), bimodal size distribution of this subset could be visualized by Image Stream (Fig. S2A). Cells with diameter within 5–8 µm range accounted for 41.83%, while cells larger than 8 µm accounted for 52.29% of events, with means of 7.06 µm ±0.48 µm and 9.27 µm ±1.14 µm, respectively. Events with diameter within 2–4 µm range were hardly detectable (3.27%) (Fig. S2A).

**Figure 2:**
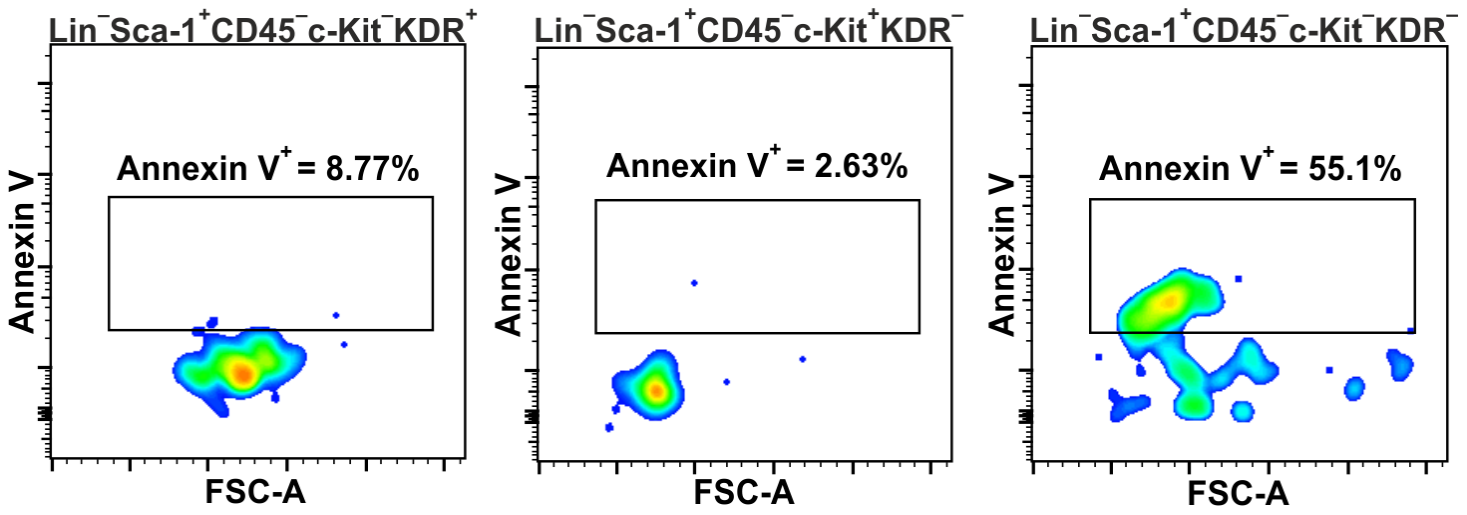
Binding of annexin V on Lin^−^Sca-1^+^CD45^−^ subpopulations. Presence of annexin V on surface of cells from Lin^−^Sca-1^+^CD45^−^ subpopulations was evaluated by flow cytometry. Majority of Lin^−^Sca-1^+^CD45^−^c-Kit^−^KDR^+^ and Lin^−^Sca-1^+^CD45^−^c-Kit^+^KDR^−^ were annexin V negative, while more than half of the Lin^−^Sca-1^+^CD45^−^c-Kit^−^KDR^−^ cells were annexin V positive.

The cell shrinkage resulting in a lower FSC values is the feature typical for cells undergoing apoptosis [23]. We tested all three Lin^−^Sca-1^+^CD45^−^ subsets for the presence of an apoptotic marker, phosphatidylserine on a cell surface, labeling them with annexin V. The c-Kit^−^KDR^+^ and c-Kit^+^KDR^−^ subsets hardly presented annexin V binding, while more than half of c-Kit^−^KDR^−^ cells were annexin V positive, showing also predominantly lower FSC values (Fig. 2).

### Lack of Oct-4 mRNA Expression in Adult Murine BM and Lin^−^Sca-1^+^CD45^−^FSC^low^ Cells

Expression of Oct-4 was demonstrated as one of molecular hallmarks of pluripotency in VSELs [6]. Given that Lin^−^Sca-1^+^CD45^−^ population is heterogeneous, we wanted to evaluate the level of Oct-4 mRNA expression in each subset.

**Figure 3:**
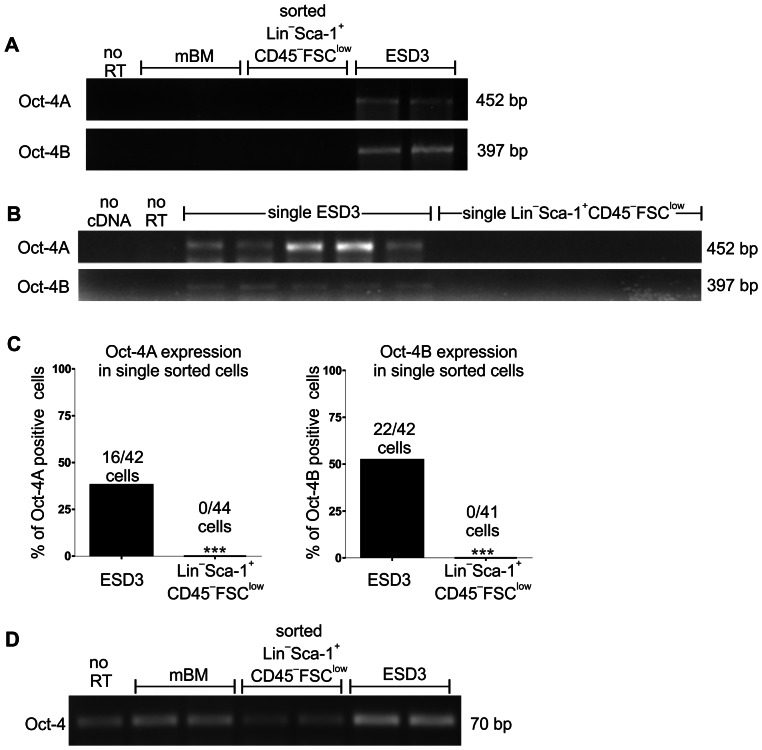
Lack of Oct-4 mRNA expression in murine bone-marrow and sorted Lin^−^Sca-1^+^CD45^−^FSC^low^ cells. (**A**) RT-PCR performed with primers used in current study [21] did not detect Oct-4A and Oct-4B mRNA in bone marrow and sorted Lin^−^Sca-1^+^CD45^−^ FSC^low^cells. (**B**) Representative analysis of Oct-4A and Oct-4B expression in single sorted Lin^−^Sca-1^+^CD45^−^FSC^low^ cells. No expression was detected. (**C**) Quantitative analysis of expression of Oct-4A and Oct-4B in single sorted Lin^−^Sca-1^+^CD45^−^ FSC^low^cells. RT-PCR did not detect Oct-4A and Oct-4B mRNA in bone marrow and sorted Lin^−^Sca-1^+^CD45^−^ FSC^low^cells. (**D**) RT-PCR performed with primers used in earlier studies [6] generated false-positive signal in no RT control, bone marrow, and sorted Lin^−^Sca-1^+^CD45^−^FSC^low^ cells. ESD3 served as a positive control. *** - p<0.001 in comparison to ESD3.

We used 2 sets of primers proposed by Mizuno and Kosaka [21] to examine Oct-4 expression. One set detects only longer isoform (Oct-4A) and second set detects also shorter (Oct-4B) splicing isoform of murine Oct-4 mRNA. Unexpectedly, qRT-PCR revealed that there is neither Oct-4A nor Oct-4B expression in sorted Lin^−^Sca-1^+^CD45^−^FSC^low^ subpopulation, despite a strong positive signal obtained from ESD3, mouse embryonic stem cell line (Fig. 3A). Accordingly, we did not detect Oct-4A and Oct-4B expression in whole murine BM (Fig. 3A). To exclude the possibility that among tested populations there were only few cells that expressed Oct-4A, what could be masked by majority of Oct-4 negative cells, we performed also a single cell RT-PCR analysis of Oct-4A and Oct-4B expression in the Lin^−^Sca-1^+^CD45^−^FSC^low^ VSELs sorted on the AmpliSpeed grid slides (Fig. 3B,C). No specific signal was detected in the no-RT and no-template controls (Fig. 3B). Oct-4A and Oct-4B mRNA was found in 38.1% (16/42) and 52.4% (22/42) of sorted ESD3 cells, respectively, while no positive cells (0/44) were detected in single-sorted Lin^−^Sca-1^+^CD45^−^FSC^low^ population (Fig. 3C).

Because our results were inconsistent with those published by Kucia and colleagues, who had demonstrated the presence of Oct-4 mRNA in BM-derived or liver-derived VSELs [6], [9], [24], we carried out an additional analysis employing primers described in the previous reports [6], [9], [24]. In this case, we were able to detect the product of expected length (70 bp) both in whole BM and in sorted VSEL population (Fig. 3D). However the same product was present in samples where no reverse transcription (RT) reaction was performed (Fig. 3D), indicating a false positive result caused by possible detection of pseudogenes. Indeed, blasting the sequences of primers with mouse genomic DNA (Primer Blast global alignment algorithm [25]) evidenced that while primers proposed by Mizuno and Kosaka [21] did not show any complementarity to genomic Oct-4 pseudogenes, those used in reports by Kucia and colleagues [6], [9], [24] bound to genomic sequence on chromosome 3, which contains Oct-4 pseudogenes [21], [26]. Moreover, they may amplify also the genomic sequence on chromosome 17 corresponding to functional Oct-4 gene. The detected few mismatches are not located within 3′ end of the primers and are unlikely to completely prevent amplification of described products on genomic DNA (gDNA). Importantly, the predicted length of product for those primers on Oct-4A cDNA is the same as for products amplified on genomic sequence.

To verify if amplification of genomic sequences may explain the false positive results, we performed the real-time PCR for Oct-4 gene on the gDNA, coupled with melt curve analysis. Melt curves for any of trace products that appeared when using Mizuno and Kosaka primers [21] were considerably different than that from positive control of ESD3-derived cDNA (Fig. S4). In contrast, primers applied by Kucia and colleagues [6], [9], [24] led to amplification of products both on gDNA template and in no-RT control, with melt curve highly similar to that from BM cDNA or from positive control (Fig. S4). Treatment of total RNA with DNase I prior to reverse transcription clearly affected the amplification of Oct-4 (Fig. S4), while did not change the generation of product from control EF-2 gene. This confirms that primers used by Kucia and co-workers [6], [9], [24] can amplify Oct-4 pseudogenes sequences carried over with genomic DNA contamination.

### Single Cell-derived Hematopoietic Colony *in vitro* Assay

Lin^−^Sca-1^+^CD45^−^ cells were reported to give rise to hematopoietic colonies *in vitro* in OP-9 co-culture assay, although hematopoietic potential of freshly isolated VSELs has not been observed [7]. Lin^−^Sca-1^+^CD45^−^c-Kit^+^KDR^−^ subpopulation, being positive for Sca-1 and c-Kit while negative for lineage markers (SKL phenotype), overlaps with classically defined hematopoietic stem cells (HSC). Therefore we used a single cell-derived hematopoietic colony test to check whether Lin^−^Sca-1^+^CD45^−^c-Kit^+^FSC^low^ subset may contain the clonogenic hematopoietic cells. Sorted cells were cultured in a chemically-defined serum-free medium to exclude the possibility of blocking the colony growth by unknown serum factors. The Lin^−^Sca-1^+^CD45^+^c-Kit^+^ population was used in parallel as a positive control. Doublets and DAPI^+^ cells were excluded in sorting strategy (Fig. S3A).

**Figure 4:**
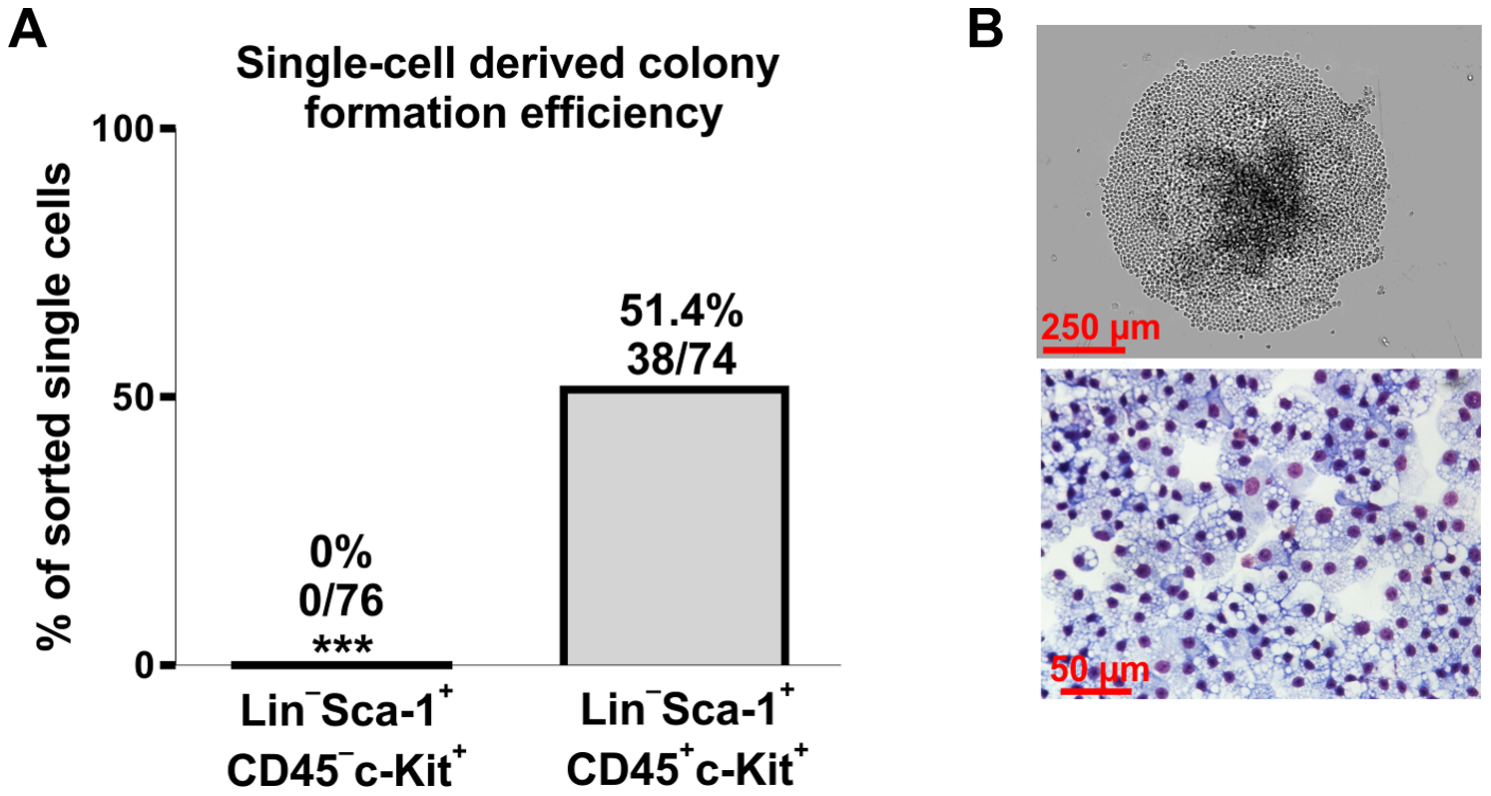
Verification of hematopoietic potential of Lin^−^Sca-1^+^CD45^−^c-Kit^+^ or Lin^−^Sca-1^+^CD45^+^c-Kit^+^ (positive control) cells by single cell-derived colony *in-vitro* assay. (**A**) Quantitative analysis of colony formation efficacy. (**B**) Example of a colony formed by Lin^−^Sca-1^+^CD45^+^cKit^+^ cells (contrast-phase microscopy and Wright’s staining) containing relatively homogenous progeny. *** - p<0.001 versus Lin^−^Sca-1^+^CD45^+^c-Kit^+^ cells.

Colonies were formed in 51.4% (38/74) of wells with single Lin^−^Sca-1^+^CD45^+^c-Kit^+^ cells (Fig. 4A). The Wright’s staining revealed the presence of progeny derived from different hematopoietic lineages within a single colony, proving the multipotency of sorted cell, although the contribution of particular cell lineages varied between the wells. There were also some colonies formed by morphologically homogenous cells, originating possibly from a more differentiated progenitor (Fig. 4B). In contrast, none of Lin^−^Sca-1^+^CD45^−^c-Kit^+^ FSC^low^ (0/76) gave rise to a colony (Fig. 4A).

**Figure 5:**
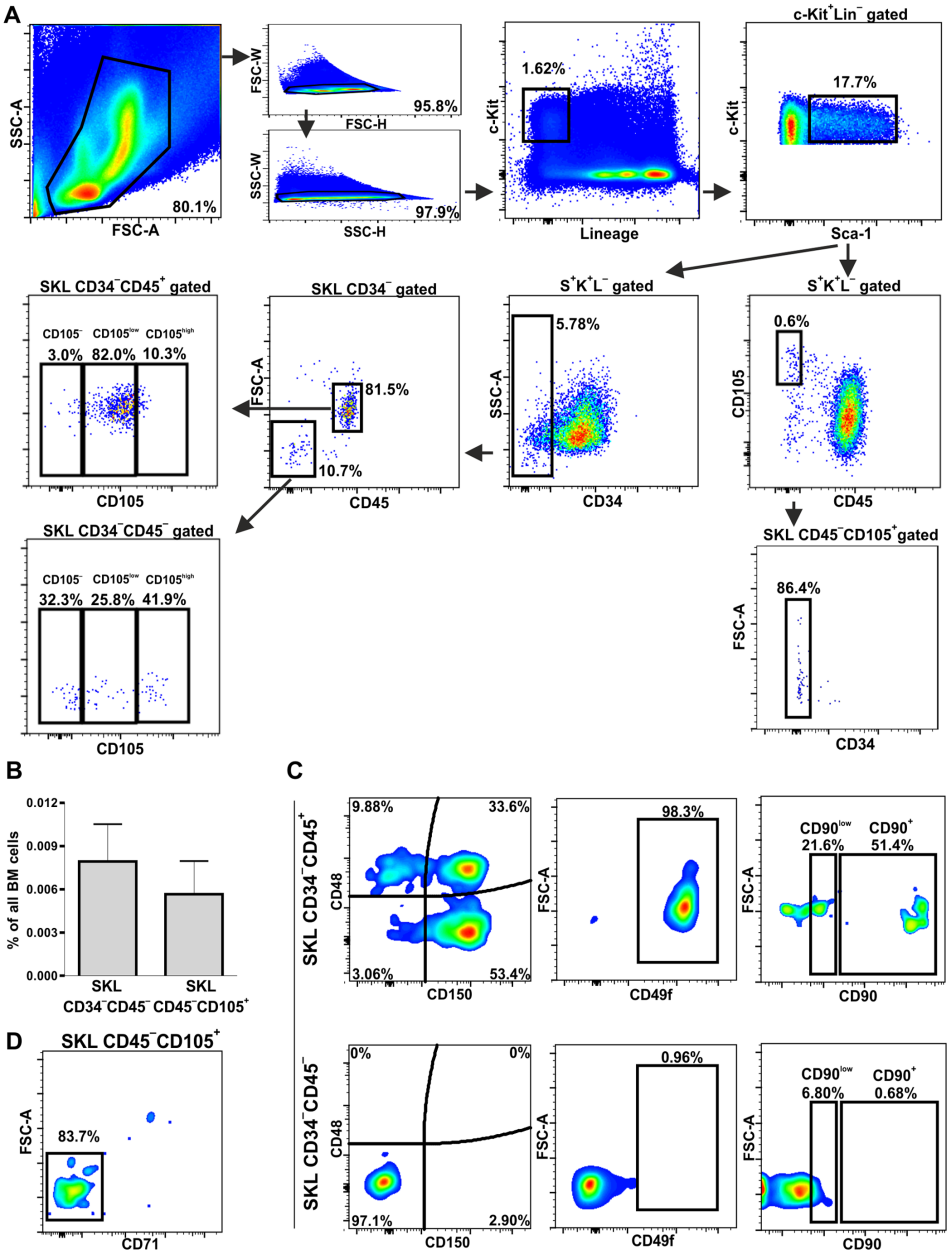
Characterization of CD45^−^ subsets within classically defined hematopoietic stem cells. (**A**) Flow cytometry analysis evidenced SKL CD34^−^CD45^−^ and SKL CD45^−^CD105^+^ cells among SKL phenotype. These populations were highly overlapping as most of SKL CD45^−^CD105^+^ were CD34 negative. (**B**) Quantitative analysis of SKL CD34^−^CD45^−^ and SKL CD45^−^CD105^+^ cells frequency in murine BM. Mean ± SD of 3–7 independent experiments. (**C**) Evaluation of CD48, CD150, CD49f and CD90 expression demonstrated lack of SLAM code markers of LT-HSC on SKL CD34^−^CD45^−^ and different marker profile when compared to SKL CD34^−^CD45^+^ cells. (**D**) Lack of CD71 expression on SKL CD45^−^CD105^+^ cells distinguished this population from erythroblasts.

Conventional gating strategy for SKL CD34^−^ cells [20], [27] revealed the presence of minor SKL CD34^−^CD45^−^subpopulation in murine bone marrow (Fig. 5A). Moreover, we were able to distinguish population defined as SKL CD45^−^CD105^+^ (Fig. 5A), thus expressing the CD105 marker, shown to enrich for LT-HSC [28], [29]. Subsequent analyzes revealed that more than 70% of SKL CD45^−^CD105^+^ are CD34 negative (Fig. 5A) showing that SKL CD34^−^CD45^−^ and SKL CD45^−^CD105^+^ are highly overlapping. Consistently, both of these subpopulations showed FSC^low^ characteristics. The frequencies of cells defined as SKL CD34^−^CD45^−^or SKL CD45^−^CD105^+^ in murine bone marrow measured by the flow cytometry were 0.0079% ±0.0026% and 0.0057% ±0.0023%, respectively (Fig. 5B), what is similar to the frequency of LT-HSC in mouse bone marrow demonstrated by the bone marrow transplant experiments [30], [31]. Interestingly, such defined SKL CD45^−^CD105^+^ subsets substantially overlapped with Lin^−^Sca-1^+^CD45^−^c-Kit^+^FSC^low^ subpopulation, with more than 70% of Lin^−^Sca-1^+^CD45^−^c-Kit^+^FSC^low^ being CD105-positive (Fig. S5).

Further phenotyping of SKL CD34^−^CD45^−^and SKL CD45^−^CD105^+^ cells demonstrated that they do not share all characteristics of LT-HSC. Analysis of the signaling lymphocyte activating molecule (SLAM) markers [32], revealed that the SKL CD34^−^CD45^−^ were CD48^−^CD150^−^, in contrast to the SKL CD34^−^CD45^+^ cells, part of which displayed the CD48^−^ CD150^+^ phenotype (Fig. 5C). Moreover, differently than SKL CD34^−^CD45^+^ population, the SKL CD34^−^CD45^−^ counterpart did not contain the CD49f-positive or CD90^hi^ cells and showed lower percentage of the CD90^low^ subset (Fig. 5C).

CD105 expression is not only a characteristic feature of LT-HSC cells [28], [29], but is also typical for erythroid precursors [33], [34], which additionally have downregulated CD45 level [35]. Therefore, expression of CD71, the erythroid marker [36], was evaluated on SKL CD45^−^CD105^+^ cells. As shown in Fig. 5D majority of SKL CD45^−^CD105^+^ cells did not have CD71, what suggested this population was distinct from CD71-positive early erythroid precursors.

**Figure 6:**
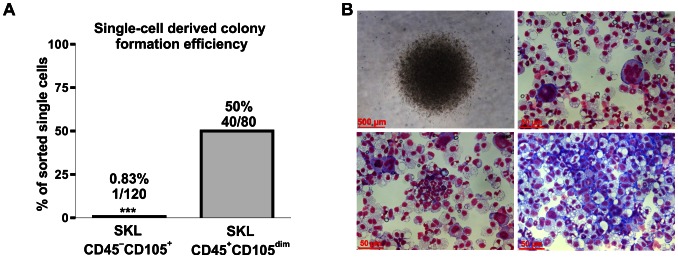
Verification of hematopoietic potential of SKL CD45^−^CD105^+^ and SKL CD45^+^CD105^dim^ cells. (**A**) Quantitative analysis of colony formation efficacy by SKL CD45^−^CD105^+^ and SKL CD45^+^CD105^dim^ (positive control) cells in single cell-derived colony *in vitro* assay. (**B**) Representative colony formed by SKL CD45^+^CD105^dim^ cells (contrast-phase microscopy and Wright’s staining) with cells derived from different hematopoietic lineages evidenced presence of multipotent hematopoietic cells among SKL CD45^+^CD105^dim^ population.

Next we checked whether the Lin^−^Sca-1^+^CD45^−^c-Kit^+^CD105^+^ cells display a clonogenic hematopoietic potential. The SKL CD45^+^CD105^dim^ population was used in parallel as positive control (CD105 is expressed on SKL CD45^+^ at lower level than on SKL CD45^−^CD105^+^, Fig. 5A, Fig. S3B). Again, in a single cell-derived hematopoietic colony *in vitro* assay the colonies were formed in 50% of wells (40/80) with single SKL CD45^+^CD105^dim^ cells (Fig. 6A,B) and all of them contained progeny derived from different hematopoietic lineages (Fig. 6B). In contrast, only 1 out of 120 sorted single SKL CD45^−^CD105^+^cells (0.83%) gave rise to a colony (Fig. 6A). This efficacy did not overcome sorting purity limits, thus we conclude that SKL CD45^−^CD105^+^ subpopulation did not show hematopoietic stem cell activity in the performed assay.

**Figure 7:**
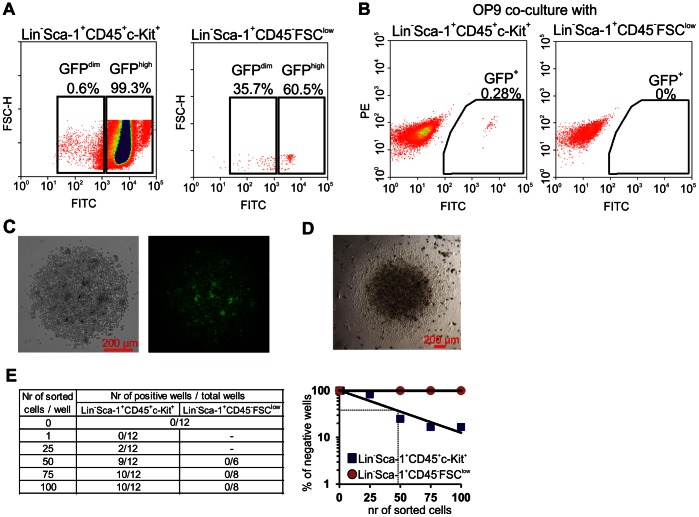
Hematopoietic differentiation of Lin^−^Sca-1^+^CD45^−^FSC^low^ and Lin^−^Sca-1^+^CD45^+^c-Kit^+^ cells after OP9 co-culture. (**A**) Lin^−^Sca-1^+^CD45^−^FSC^low^ isolated from the BM of C57BL/6-Tg(UBC-GFP)30Scha/J mice presented lower GFP expression comparing to Lin^−^Sca-1^+^CD45^+^c-Kit^+^ cells from this mouse strain. (**B**) Lin^−^Sca-1^+^CD45^−^FSC^low^ isolated from the C57BL/6-Tg(UBC-GFP)30Scha/J mice lost GFP expression after 4 days co-culture with OP9 cells. In contrast, Lin^−^Sca-1^+^CD45^+^c-Kit^+^ sustained GFP expression after the co-culture. (**C**) Representative colony formed by single Lin^−^Sca-1^+^CD45^+^c-Kit^+^cell that was sorted from co-culture with OP9. (**D**) Representative colony formed by single Lin^−^Sca-1^+^CD45^+^c-Kit^+^ co-cultred with OP9 after transferring to hematopoietic differentiation media. (**E**) Limiting dilution analysis of colony forming cells among Lin^−^Sca-1^+^CD45^−^FSC^low^ and Lin^−^Sca-1^+^CD45^+^c-Kit^+^ co-cultured with OP9 cells.

In the last step of analysis we verified if hematopoietic potential of any subpopulations within Lin^−^Sca-1^+^CD45^−^FSC^low^ fraction could be induced by co-culture with OP9 cells as suggested before [7]. To this aim, 1,000 Lin^−^Sca-1^+^CD45^−^FSC^low^ or Lin^−^Sca-1^+^CD45^+^c-Kit^+^ cells from BM of GFP^+^ mice were sorted onto well of 96-well plate with confluent OP9 culture. After 4 days of co-culture, the GFP^+^ cells from the co-culture were planned to be sorted for a single cell-derived hematopoietic colony assay as described above. However, Lin^−^Sca-1^+^CD45^−^FSC^low^ cells expressed GFP at lower levels than Lin^−^Sca-1^+^CD45^+^c-Kit^+^ counterparts during the sort for OP9 co-culture (Fig. 7A) and completely lost GFP expression after 4 days of co-culture with OP9 (Fig. 7B), what made testing their hematopoietic potential by single-cell method impossible. In contrast, the Lin^−^Sca-1^+^CD45^+^c-Kit^+^ population sustained high GFP expression after co-culture with OP9 (Fig. 7A,B), and 35.5% (38 of 107 sorted wells) of single sorted GFP^+^ cells formed hematopoietic colonies (Fig. 7C).

The observed decline of GFP expression in Lin^−^Sca-1^+^CD45^−^FSC^low^ fraction may be caused by death of the cells during co-culture with OP9 feeders or by deactivation of the promoter driving the GFP expression in Lin^−^Sca-1^+^CD45^−^FSC^low^ population. To exclude the latter possibility, we sorted from 100 to 1 Lin^−^Sca-1^+^CD45^−^FSC^low^ or Lin^−^Sca-1^+^CD45^+^c-Kit^+^ cells per well of 96-well plate with confluent OP9 culture and after 5 days all cells, including OP9 cells, were transferred to hematopoietic differentiation media. The limiting dilution analysis revealed that approximately 1 per 48 sorted Lin^−^Sca-1^+^CD45^+^c-Kit^+^ cells displayed the colony forming ability (Fig. 7D,E) while no colony growth was observed in Lin^−^Sca-1^+^CD45^−^FSC^low^ group (Fig. 7E). This indicates that Lin^−^Sca-1^+^CD45^−^FSC^low^ population did not possess hematopoietic cells with clonogenic potential even after priming with OP9 cells.

### Viability of Lin^−^Sca-1^+^CD45^−^c-Kit^+^CD105^+^

**Figure 8:**
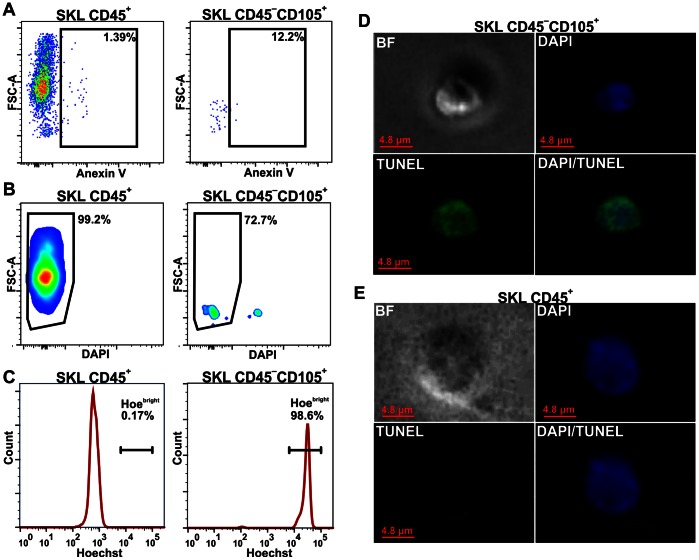
Viability of SKL CD45^−^CD105^+^ and SKL CD45^+^ cells. (**A**) SKL CD45^−^CD105^+^ cells are mainly annexin V negative. (**B**) Most of the SKL CD45^−^CD105^+^ cells possess an integral membrane, however this population is enriched in non-viable cells without membrane integrity when compared to SKL CD45^+^. (**C**) The SKL CD45^−^CD105^+^ cells stained highly with Hoechst 33342 nuclear dye. (**D**) TUNEL analysis of sorted subset revealed chromatin fragmentation in SKL CD45^−^CD105^+^ cells. (**E**) Chromatin fragmentation was not found in SKL CD45^+^ population. *** - p<0.001 versus SKL CD45^+^CD105^dim^ cells.

Lack of colony-forming capacities of the SKL CD45^−^CD105^+^ cells prompted us to more detailed investigation of their viability. Backgating has revealed that this population, despite being Annexin-V negative (Fig. 8A), was enriched in DAPI^+^ events, when compared with SKL CD45^+^ fraction. Nevertheless, most of these cells (>70%) were DAPI-negative, indicating an intact cell membrane (Fig. 8B). Moreover, DAPI^+^ cells were excluded during the single cell sorting and were not taken for hematopoietic assay (Fig. S3). Interestingly, all SKL CD45^−^CD105^+^ cells stained highly with Hoechst 33342, with the signal over one order of magnitude higher than that from SKL CD45^+^ subset (Fig. 8C). This excluded a possibility that the difference resulted from a distinct cell cycle status. Instead, increased permeability for Hoechst may be one of early apoptosis symptoms [37]. Therefore, we sorted 50 cells on the slide glass and analyzed them using TUNEL assay [38] (Fig. 8D, 8E). It turned out that 83.3% of SKL CD45^−^CD105^+^ cells showed chromatin fragmentation, while all control SKL CD45^+^ were TUNEL-negative.

Summing up, SKL CD45^−^CD105^+^ cells had intact cell membrane, did not bind annexin V, but showed increased permeability for Hoechst 33342 dye and displayed chromatin fragmentation, which altogether indicates that the population most possibly consisted of early apoptotic cells.

### Origin of SKL CD45^−^CD105^+^ Population in Mouse BM

Given that Lin^−^Sca-1^+^CD45^−^c-Kit^+^CD105^+^ events seemed to be early apoptotic cells, we aimed to determine the possible ancestor population that they can derive from.

**Figure 9:**
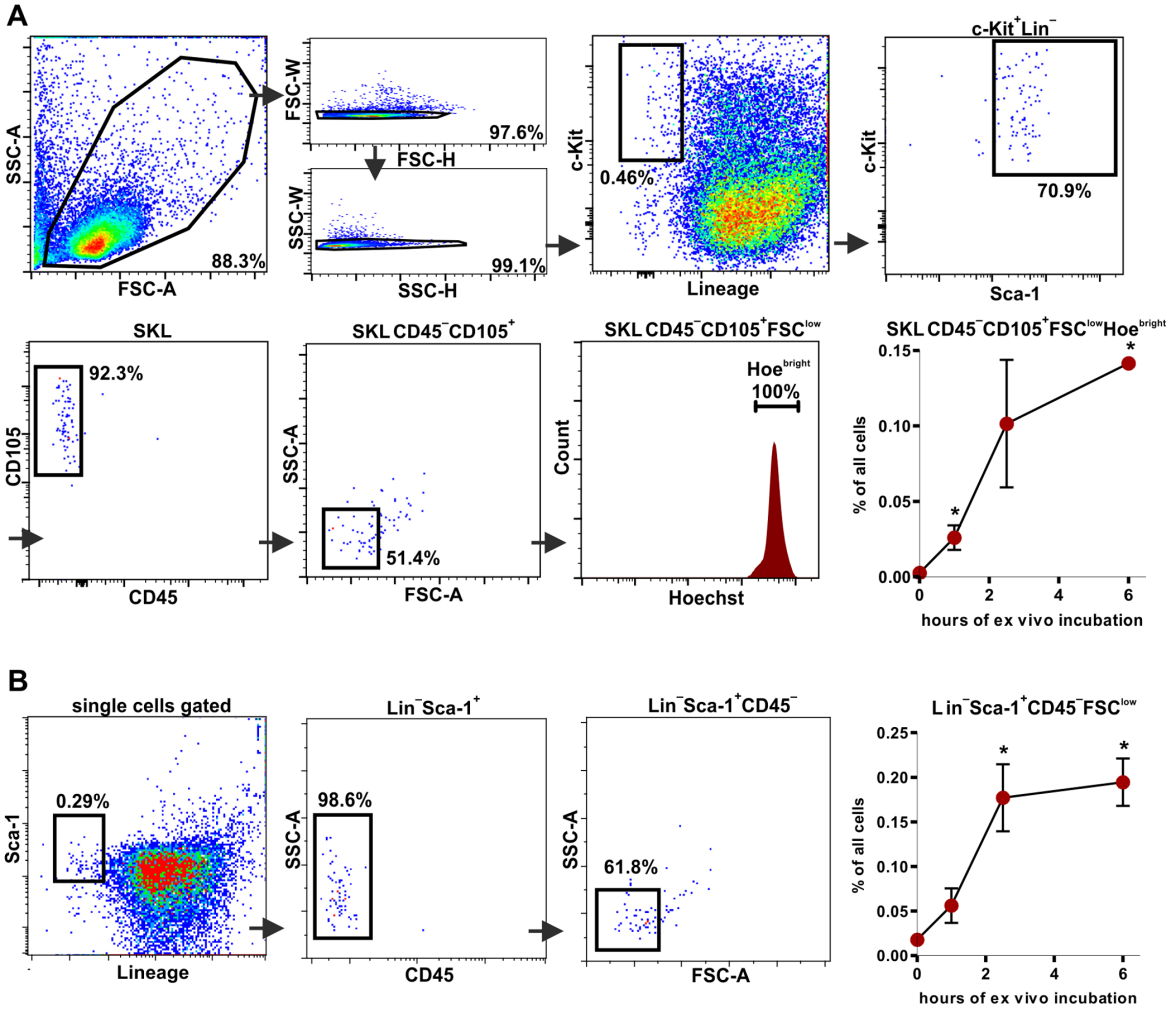
Phenotype of nuclei expelled from *ex vivo* cultured erythroblasts. (**A**) Gating strategy and analysis of number of events with the SKL CD45^−^CD105^+^FSC^low^Hoe^high^ phenotype during *ex vivo* enucleation process. (**B**) Gating strategy and analysis of number of events with the Lin^−^Sca-1^+^CD45^−^FSC^low^ phenotype, during *ex vivo* enucleation process.* - p<0.05 vs. t = 0 h.

Features such as small size, high staining with nuclear dye and integral membrane resembles characteristics of nuclei expelled from erythroblasts during erythropoiesis (so called pyrenocytes) [16], [17]. Yet, there are no data indicating if the expelled nuclei could share SKL CD45^−^CD105^+^ marker profile. To verify if the origin of BM Lin^−^Sca-1^+^CD45^−^c-Kit^+^CD105^+^ cells can be associated with enucleation process, we sorted Ter119^+^CD45^−^ erythroblasts from bone marrow and incubated them *ex vivo* for 6 hours in 37°C to induce the enucleation process [16]. During time of incubation the kinetics of formation of small SKL CD45^−^CD105^+^ events, highly stained with Hoechst 33342, was evaluated (Fig. 9A).

All gates were set in the same manner as for the SKL CD45^−^CD105^+^ cells in whole bone marrow samples (Fig. 9A). Immediately after erythroblast sorting the SKL CD45^−^CD105^+^ events were barely detectable. However, their number significantly increased with the incubation time (Fig. 9A). These data suggest that nuclei expelled from the erythroblasts can transiently present SKL CD45^−^CD105^+^ phenotype. Accordingly, we observed similar increase in a number of events defined as classic VSELs (Lin^−^Sca-1^+^CD45^−^FSC^low^) (Fig. 9B). Thus, it is likely that SKL CD45^−^CD105^+^ and Lin^−^Sca-1^+^CD45^−^FSC^low^ populations isolated from the bone marrow can include the nuclei released during erythropoiesis.

## Discussion

In the present study we investigated the mouse bone-marrow derived cells with Lin^−^Sca-1^+^CD45^−^ phenotype and small size. Such cells, named VSELs (very small embryonic-like cells), were described in 2006 by Kucia and colleagues as a homogenous population of pluripotent cells [6] or population enriched in pluripotent cells [9], expressing Oct-4 marker, and acting among others as precursors of long-term repopulating hematopoietic stem cells (LT-HSC) [7]. Our results indicate, however, that the mouse BM population defined as Lin^−^Sca-1^+^CD45^−^ FSC^low^ is heterogeneous and does not express Oct-4.

Using two more markers, c-Kit and KDR, we could distinguish three subsets within the Lin^−^Sca-1^+^CD45^−^ cells: c-Kit^−^KDR^+^, c-Kit^−^KDR^−^, and c-Kit^+^KDR^−^. Events positive for both, c-Kit and KDR, detected in some samples, were too rare to allow reliable measurement by flow cytometry. Because c-Kit^−^KDR^+^ fraction consisted of cells with relatively big diameter, it did not fulfill VSEL size criterion and was excluded from further analysis. On the other hand, the entire c-Kit^+^KDR^−^ population and part of c-Kit^−^KDR^−^ subset contained far smaller cells, in compliance with definition of VSELs (Fig. 1C,D, Fig. S2A). Among them, c-Kit^−^KDR^−^ cells with lower FSC values were apoptotic, as they bound annexin V. We are aware that RBC-derived microvesicles (RMV) released during erythrocyte lysis may transfer phosphatidylserine (PS) to the surface of other cells, resulting in false-positivity for annexin V and erythrocyte marker glycophorin A [39]. However, it is not likely to be a case, as cells with erythrocyte marker Ter119^+^ (glycophorin A-associated protein) [40] were excluded from analysis together with lineage committed populations. Additionally, other subpopulations present in the same samples were annexin V negative and there are no data suggesting selective binding of RMV to Lin^−^Sca-1^+^CD45^−^c-Kit^−^KDR^−^ cells. Thus is seems that Lin^−^Sca-1^+^CD45^−^c-Kit^−^KDR^−^FSC^low^ cells were indeed apoptotic. Distinguishing a tentative stem cells fraction within this population, based on small size only, is not possible in reproducible way, unless discovery of next marker, better defining viable cells. Therefore, our further work was focused on Lin^−^Sca-1^+^CD45^−^c-Kit^+^KDR^−^ subset.

The Lin^−^Sca-1^+^CD45^−^c-Kit^+^KDR^−^ cells were annexin V negative, and showed homogeneity of the size distribution, with diameter of 6.8±2.4 µm, which was similar to that of erythrocytes (6.2±0.6 µm) and significantly smaller than in control Lin^−^Sca-1^+^CD45^+^c-Kit^+^ hematopoietic progenitors (11.4±0.7 µm). Inconsistencies in description of the exact size of VSELs in previous reports make them difficult for a direct comparison with our data. Zuba-Surma and co-workers calculated the murine BM-derived VSEL size as 3.63±0.27 µm and Lin^−^Sca-1^+^CD45^+^ HSCs as 6.58±1.09 µm [41]. Both these values are smaller than measured by us, although the HSC:VSEL size ratio is similar (1.8 versus 1.7 in previous and current report, respectively). In fact, in our hands the analysis of very small events (with diameter of 2–4 µm) using the ImageStream system (applied by Zuba-Surma and co-workers) did not allow for reliable detection of viable cells (Fig. S2B). Importantly, in another report by the same researchers, size of Lin^−^Sca-1^+^CD45^−^ cells isolated from different organs varied profoundly ranging from 3.63±0.27 µm for VSELs obtained from bone marrow to 6.81±0.09 µm, 6.90±0.29 µm, and 8.40±0.17 µm for VSELs obtained from murine spleen, heart and liver, respectively [9].

As a principle for VSEL analysis, a population of events with diameter of 2–10 µm is used, based on the size calibration beads [7], [9], [10]. It also should be taken into consideration that fixation, and following preparation of cells for transmission electron microscopy or for intracellular staining, does affect cell size [42], what may explain a difference between our measurement (done on non-fixed cells) and earlier analyses performed on fixed cells [6], [9]. In addition, assessing the real diameter of cells using the synthetic beads as a size reference is of limited accuracy, as the light scattered along the laser axis (FSC) is not solely dependent on cell size, but also on the refractive index which can be different for cells and beads, and which can be affected by physiological state of a cell [43]. For example, dead cells typically have a lower refractive index due to leaky outer membranes, give lower FSC signals, and thus appear smaller than healthy cells. Concluding, it is highly probable that c-Kit^+^KDR^−^ subset of Lin^−^Sca-1^+^CD45^−^ cells with their relative small size, was included within VSELs population in previous studies.

Noteworthy, the heterogeneity of murine Lin^−^Sca-1^+^CD45^−^ cells was recently shown with respect to the expression of platelet-derived growth factor-α receptor (PDGFR-α), which is predominantly absent on SKL cells [44]. The PDGFR-α positive fraction was suggested as overlapping with CD105 positive multipotent mesenchymal precursors identified in murine bone marrow [19]. These cells, however, are different from our subpopulation, as they do not express c-Kit [19].

VSELs are described by their discoverers as the cells expressing Oct-4, what can be a feature characteristic for pluripotent cells [6], [7], [9], [24], [45]–[47]. Oct-4 transcription factor forms two splicing forms: whilst Oct-4A has been confirmed as the isoform responsible for maintaining the embryonic stem cell identity, Oct-4B localizes mainly in the cytoplasm of pluripotent cells, various non-pluripotent cells, and somatic cells, fails to confer ES cell self-renewal and pluripotency, and is thought to be involved in cell stress responses [48]–[50]. Recent study demonstrated that mouse Oct-4B can be translated into three distinct isoforms (Oct4B-247aa, Oct4B-190aa, and Oct4B-164aa) and, in contrast to Oct-4A, it is not a functional factor in process of reprogramming somatic cells to induced pluripotent stem cells (iPSC) [50]. Therefore, in research regarding pluripotency, it is necessary to distinguish Oct4A and Oct4B variants.

Importantly, using the primers recognizing specifically a functional Oct-4A [21] we were not able to detect the Oct-4A expression at mRNA level either in whole murine bone marrow or in sorted Lin^−^Sca-1^+^CD45^−^FSC^low^ cells. We applied both qRT-PCR in RNA isolated from sorted Lin^−^Sca-1^+^CD45^−^FSC^low^ subpopulation as well as qRT-PCR in the sorted single Lin^−^Sca-1^+^CD45^−^FSC^low^ cells, with the same outcome: no specific product for Oct-4A or Oct-4B was detected, despite strong specific signals from ESD3 murine embryonic cells used as a positive control. Our observation is consistent with studies showing the lack of Oct-4 expression in bone marrow of Oct-4-GFP transgenic mice [51], and with observation that genetic ablation of Oct-4 in several tissues did not affect homeostasis or regeneration capacity in adult mice [52].

We suppose that disparity between current and earlier analyzes of Oct-4 expression in VSELs can result from application of different primer sequences. False-positive findings caused by detection of Oct-4 pseudogenes with non-specific primers have already been evidenced [18], [53], [54]. Here we demonstrated that primers used in the first paper by Kucia and co-workers [6] and then in the other reports [9], [24] may easily produce false positive results. Using them, we obtained an Oct-4 signal of expected length both in whole bone marrow and in sorted Lin^−^Sca-1^+^CD45^−^FSC^low^ VSELs, however, the product was transcribed from a pseudogene template. This false-positive reaction was not effectively prevented by DNase I treatment, what is in agreement with data described by Wang and Dai [55].

In several studies published by the same group, the Oct-4 expression in VSELs was demonstrated with additional methods – RT-PCR using re-designed primers [7], [9], [46], flow cytometry and immunohistochemistry for Oct-4 protein detection [9], [46] or analysis of Oct-4 promoter methylation [46]. Nevertheless, re-designed primers used in these studies [7], [9], [46] can still recognize both Oct-4A and Oct-4B isoforms [NM013633.3 and NM001252452.1] giving the products of the same length (51 bp). These primers allowed us to detect strong expression of Oct4 in iPSC but did not give a specific signal in our samples of total bone marrow or sorted Lin^−^Sca-1^+^CD45^−^FSC^low^ cells (data not shown). Similarly, Affymetrix microarrays used for global transcriptome profiling [47] is supposed to detect both Oct-4A and Oct-4B [53]. On the other hand, it is very difficult or even impossible to distinguish Oct-4A and Oct-4B proteins with antibodies currently available [53]–[56]. Although Oct-4A is present mostly in nucleus and Oct-4B preferentially localizes to cytoplasm, the localization itself cannot be treated as isoform marker, because Oct-4B can also display nuclear translocation [53], [55]. Interestingly, Oct-4 was found by another group in a low number of cells throughout the adult human pancreas [57]. The cells were very small (1.5–3 µm) and could be described as VSELs, but Oct-4 staining was visible only in cytoplasm [57]. Finally, analysis of methylation of Oct-4 promoter may indicate an active transcription of the gene but is not clear whether it can reveal the specific expression of Oct-4A. Altogether, it seems that the previous reports, which show Oct-4 in murine VSELs, cannot confirm the presence of Oct-4A isoform. Thus, the expression of functional Oct-4 in VSELs is still a matter of debate, while our results showed the absence of either Oct4A or Oct4B mRNA in murine bone marrow-derived Lin^−^Sca-1^+^CD45^−^FSC^low^ cells. Noteworthy, similar conclusion was driven recently from the study on human VSELs performed by Alt and co-workers [15]. The level of Oct4 signal, detected with Affymetix microarray probes (which bind both to functional gene and pseudogenes), was similar in VSELs and in B-cells used as negative control, and was significantly lower than in iPS or embryonic stem cells [15].

Lin^−^Sca-1^+^CD45^−^c-Kit^+^FSC^low^ subset of VSELs fulfills the SKL (Sca-1^+^c-Kit^+^Lin-) characteristics, so we tested whether it may contain cells with a hematopoietic stem cell potential. Single-cell assay performed in serum-free, chemically-defined medium showed that this subset, in contrast to Lin^−^Sca-1^+^CD45^+^c-Kit^+^ counterpart, did not give rise to hematopietic colonies. This result confirms earlier observations, that freshly isolated Lin^−^Sca-1^+^CD45^−^ VSELs do not show hematopoietic potential, but require co-culture with OP9 cells to acquire the HSC-like features [7]. However, it should be stressed that the colony growth observed after co-culture of VSELs with OP9 cells was not demonstrated for single cells, but for 10,000 cells seeded to each well [7]. As recently pointed out [15], the growth of hematopoietic colonies might result from inherited sorting impurities and potential presence of Lin^−^Sca-1^+^CD45^+^ cells in the populations studied, especially when cells of different size (2–10 µm) [7] were harvested.

Therefore, we examined if Lin^−^Sca-1^+^CD45^−^FSC^low^ fraction contains any cells with hematopoietic potential after priming with OP9 co-culture by applying experimental scheme that reduces risk of contamination caused by sorting impurities. However, Lin^−^Sca-1^+^CD45^−^FCS^low^ cells isolated from GFP-expressing mice lost GFP expression after 4 days of co-culture with OP9 making single cell analysis impossible, in contrast to classical hematopoietic stem and progenitor cells population (Lin^−^Sca-1^+^CD45^+^c-Kit^+^). Next experiment that included higher number of cells and limiting dilution analysis also revealed no cell with clonogenic potential among aliquots of 100 Lin^−^Sca-1^+^CD45^−^FCS^low^ cells.

These results suggest that murine Lin^−^Sca-1^+^CD45^−^FSC^low^ VSEL population does not contain cells with hematopoietic potential. The same conclusion was drawn from the study, where human VSELs did not generate hematopoietic colonies either in stroma-supported or stroma-free cultures, and were described as dysfunctional cells with karyotypic abnormalities [15]. Although lack of proof is not a proof of absence, our results appear to support the recent papers questioning VSELs as pluripotent cells with hematopoietic potential [15], [44], [58].

In next step we examined if Lin^−^Sca-1^+^CD45^−^c-Kit^+^FSC^low^ cells can be included during regular gating scheme into the LT-HSC fraction [20], [27]. Noticeably, we found that among SKL CD34^−^ subpopulation, the CD45 negative cells can be detected (SKL CD34^−^CD45^−^), but they did not accomplish all SLAM code criteria, established for LT-HSC [32]. Yet another marker defining the long-term repopulating stem cells, CD105 (endoglin) [28], [29], was highly expressed within SKL CD34^−^CD45^−^ fraction. Interestingly, although earlier reports on VSELs indicated that these cells are CD105 negative [45] our analysis demonstrate that SKL CD45^−^CD105^+^ subpopulation, apart from showing some common antigens with LT-HSC, overlapped significantly with the Lin^−^Sca-1^+^CD45^−^c-Kit^+^FSC^low^ cells. Therefore we repeated the single-cells assay to check the hematopoietic potential of this fraction. Again, in contrast to the SKL CD45^+^CD105^dim^ counterpart, the SKL CD45^−^CD105^+^ cells did not exhibit hematopoietic activity. Thorough analyzes of their viability revealed that SKL CD45^−^CD105^+^ cells display early apoptotic features, with still integral membrane (DAPI negative), without phosphatidylserine exposure (annexin V negative), but already with chromatin fragmentation process started (TUNEL positive), what explains negative results in the performed hematopoietic assays.

We noticed that several phenotypic features of SKL CD45^−^CD105^+^ events, namely the small size, high staining with nuclear dye, and integral membrane, can also characterize the nuclei expelled from erythroblasts [16]. What is more, due to differential protein sorting during erythroblast enucleation the Ter119 antigen is partitioned predominantly to the reticulocyte, and is barely detectable in extruded nuclei [59]–[62]. Similarly decreased expression was demonstrated for CD71 [62]. Our *ex vivo* analysis of the enucleation process of purified erythroblasts evidenced for the first time that some expelled nuclei possess, at least transiently, the SKL CD45^−^CD105^+^ or Lin^−^Sca-1^+^CD45^−^FSC^low^ phenotypes. Obviously, nuclei expelled from erythroblasts are heterogenous and can be also annexin V positive, as was shown by Yoshida and co-workers in splenic erythroblasts [16]. For example, in the study on primitive erythropoiesis, the expelled nuclei presented mixed phenotype according to annexin V binding (49% 7AAD^−^AnnexinV^−^, 35% 7AAD^−^AnnexinV^+^) [17]. Keeping in mind that enucleation involves dynamic changes, resulting in heterogeneous phenotype of expelled nuclei [17], it is justifiable to suppose that expelled nuclei may only transiently show the SKL CD45^−^CD105^+^ or Lin^−^Sca-1^+^CD45^−^ characteristics. Nevertheless, our results suggest that those populations isolated from the bone marrow may be contaminated with remnants of erythropoiesis.

VSELs were claimed to localize within side-population [6], what can support their viability and functional potential. However, in routine experiments they are identified by immunophenotyping only, as Lin^−^Sca-1^+^CD45^−^FSC^low^ cells. In such analysis contamination of desired population with different events, is very possible. Moreover, equivalent phenotyping of human VSELs (Lin^−^CXCR4^+^CD45^−^ FSC^low^) gave a population containing the cells highly stained with Hoechst 33342. This was interpreted as possible binuclearity, tetraploidy, or an unusual chromatin conformation in VSELs [15]. Our experiments suggest that similar effect might also result from contamination with pyrenocytes.

Altogether, our study does not support the hypothesis that cells of Lin^−^Sca-1^+^CD45^−^FSC^low^ phenotype represent a pluripotent population. In our hands these cells do not express pluripotent marker Oct-4A and do not show a hematopoietic potential in the single cell colony formation assay or after co-culture with OP9 cells. We found that Lin^−^Sca-1^+^CD45^−^FSC^low^ population is heterogenous, enriched in early apoptotic cells, and can be potentially contaminated with the nuclei expelled from the erythroblasts. Thus, although this bone marrow-derived fraction potentially can include some multipotent cells, any definitive conclusions on their properties and potency require establishment of precise immonophenotyping, purification and propagation protocols.

## Supporting Information

Figure S1 Gating strategy for ImageStream analysis.
(TIF)

Figure S2 (**A**) Representative analysis of size distribution in Lin^−^Sca-1^+^CD45^−^c-Kit^−^ cell population. (**B**) Representative pictures showing morphology of Lin^−^Sca-1^+^CD45^−^c-Kit^+^ cells. ImageStream System.
(TIF)

Figure S3 Sorting protocol for the single cell-derived colony assay. **(A)** Sorting strategy of collecting Lin^−^Sca-1^+^CD45^−^c-Kit^+^FSC^low^ and Lin^−^Sca-1^+^CD45^+^c-Kit^+^ cells. **(B)** Sorting strategy of collecting SKL CD45^−^CD105^+^ and SKL CD45^+^CD105^dim^ cells.
(TIF)

Figure S4 Analysis of melt curves of products amplified with primers used in earlier studies (Oct-4) [6], [9], [24] and with primers used in the current study (Oct-4A) [21] in sorted Lin^−^Sca-1^+^CD45^−^FSC^low^, bone marrow, and ESD3 (positive control) cDNAs, or in no RT and gDNA samples (negative controls). Primers used in earlier studies generated similar melt curves in all samples. Treatment of total RNA with DNase I prior to reverse transcription affected the amplification of Oct-4. Any trace product amplified in negative controls or tested samples with primers used in the current study presented clearly different melt curve than that amplified on ESD3 cDNA.
(TIF)

Figure S5 Flow cytometry analysis showing that SKL CD45^−^CD105^+^ subset significantly overlaps with Lin^−^Sca-1^+^CD45^−^c-Kit^+^FSC^low^ subpopulation, with more than 70% of Lin^−^Sca-1^+^CD45^−^c-Kit^+^FSC^low^ being CD105-positive.
(TIF)
