# Supplementary figures and images for: Murine Bone Marrow Lin−Sca-1+CD45− Very Small Embryonic-Like (VSEL) Cells Are Heterogeneous Population Lacking Oct-4A Expression
Source: PLoS One. 2013 May 17;8(5):e63329. doi: 10.1371/journal.pone.0063329 (PMC3656957; doi:10.1371/journal.pone.0063329)

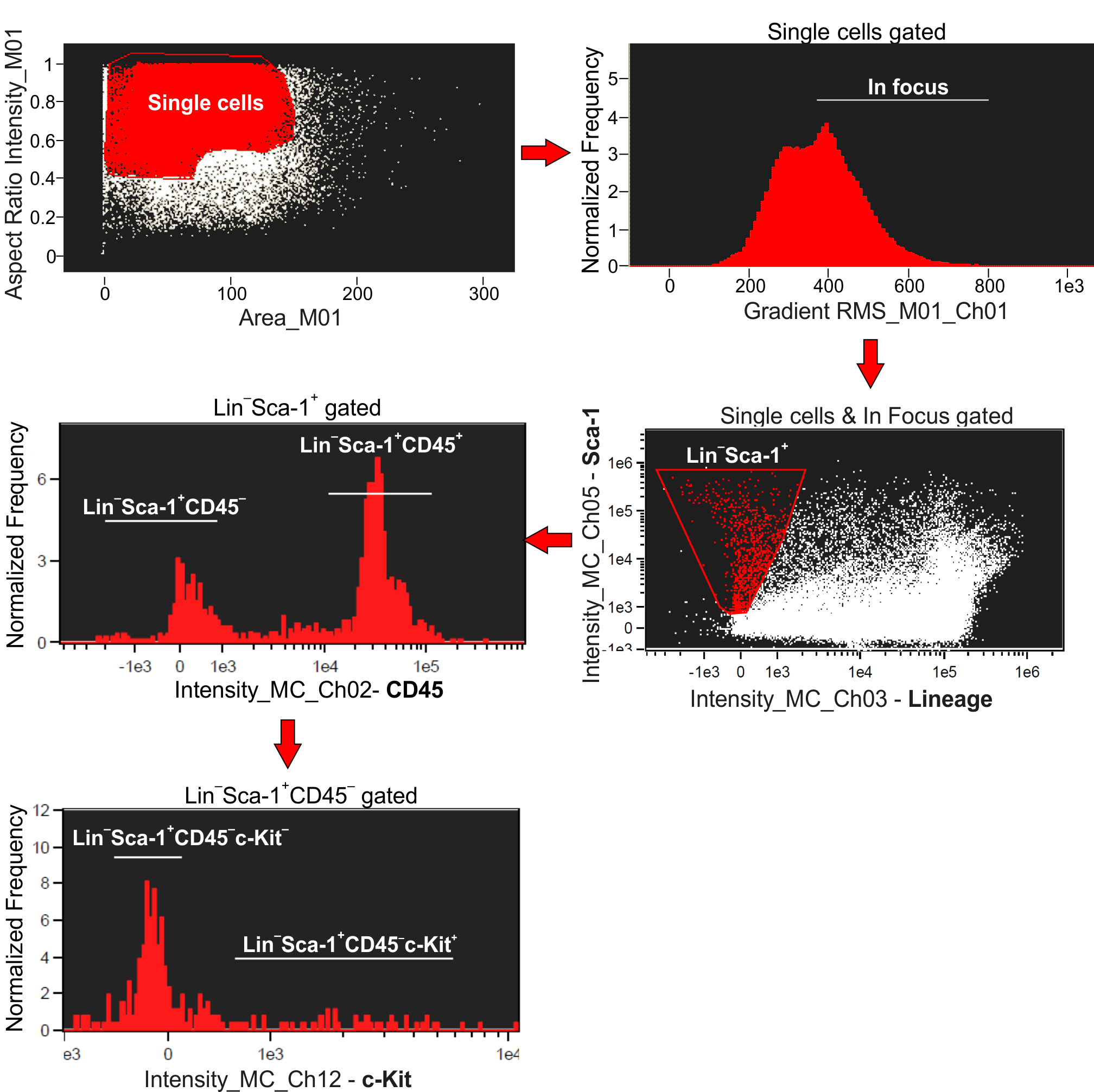

Supplement: Figure S1 — Gating strategy for ImageStream analysis. (TIF) [file pone.0063329.s001.tif]

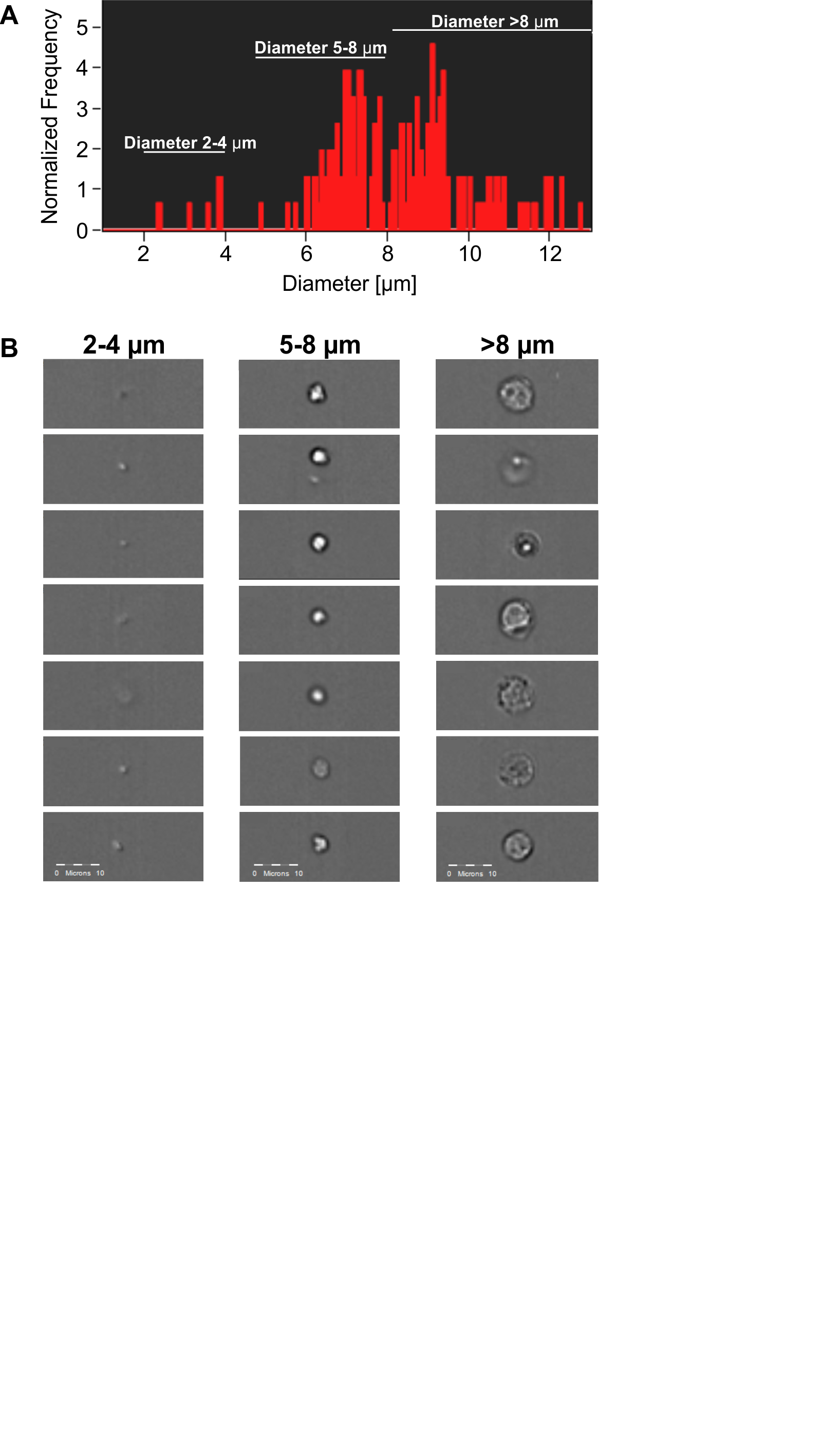

Supplement: Figure S2 — (A) Representative analysis of size distribution in Lin−Sca-1+CD45−c-Kit− cell population. (B) Representative pictures showing morphology of Lin−Sca-1+CD45−c-Kit+ cells. ImageStream System. (TIF) [file pone.0063329.s002.tif]

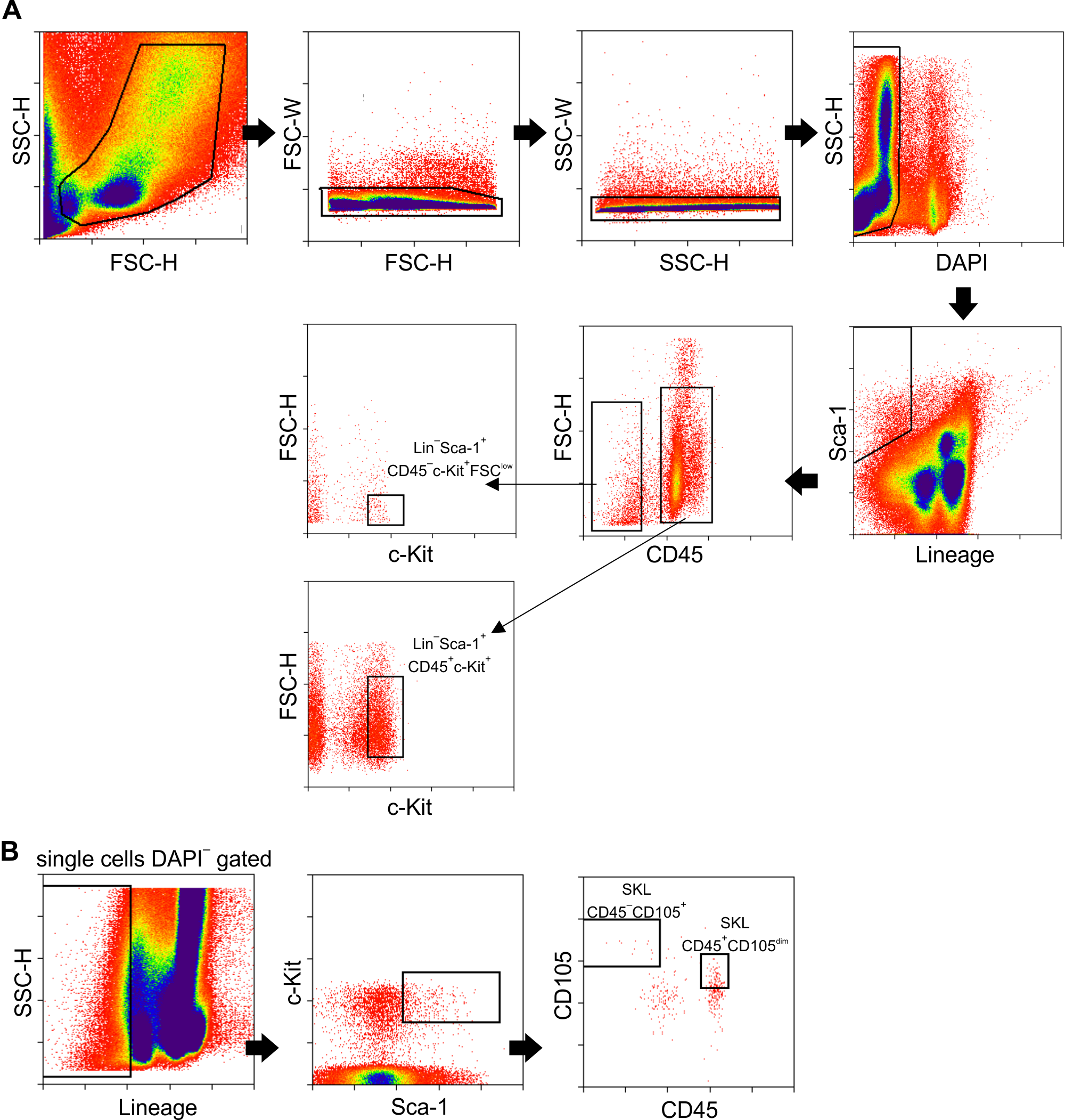

Supplement: Figure S3 — Sorting protocol for the single cell-derived colony assay. (A) Sorting strategy of collecting Lin−Sca-1+CD45−c-Kit+FSClow and Lin−Sca-1+CD45+c-Kit+ cells. (B) Sorting strategy of collecting SKL CD45−CD105+ and SKL CD45+CD105dim cells. (TIF) [file pone.0063329.s003.tif]

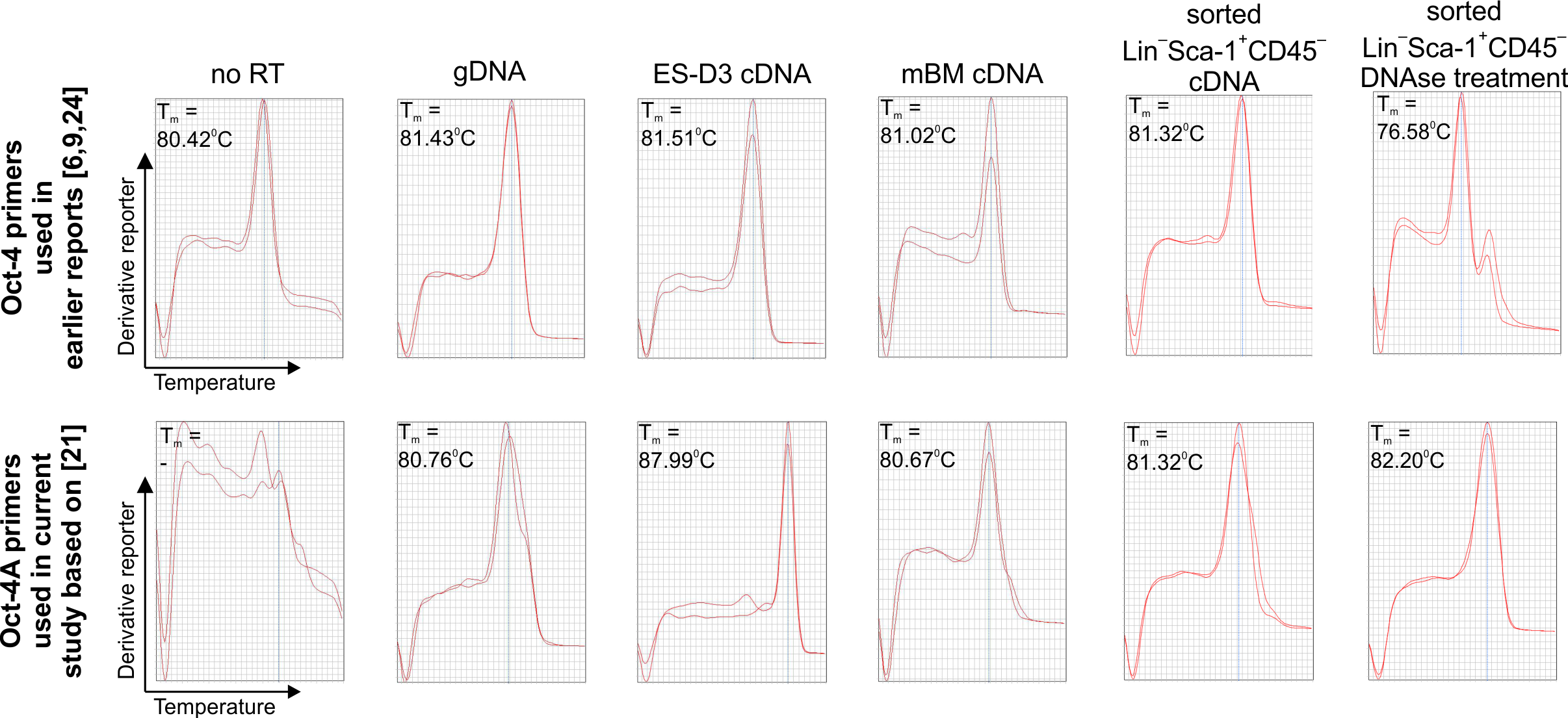

Supplement: Figure S4 — Analysis of melt curves of products amplified with primers used in earlier studies (Oct-4) [6], [9], [24] and with primers used in the current study (Oct-4A) [21] in sorted Lin−Sca-1+CD45−FSClow, bone marrow, and ESD3 (positive control) cDNAs, or in no RT and gDNA samples (negative controls). Primers used in earlier studies generated similar melt curves in all samples. Treatment of total RNA with DNase I prior to reverse transcription affected the amplification of Oct-4. Any trace product amplified in negative controls or tested samples with primers used in the current study presented clearly different melt curve than that amplified on ESD3 cDNA. (TIF) [file pone.0063329.s004.tif]

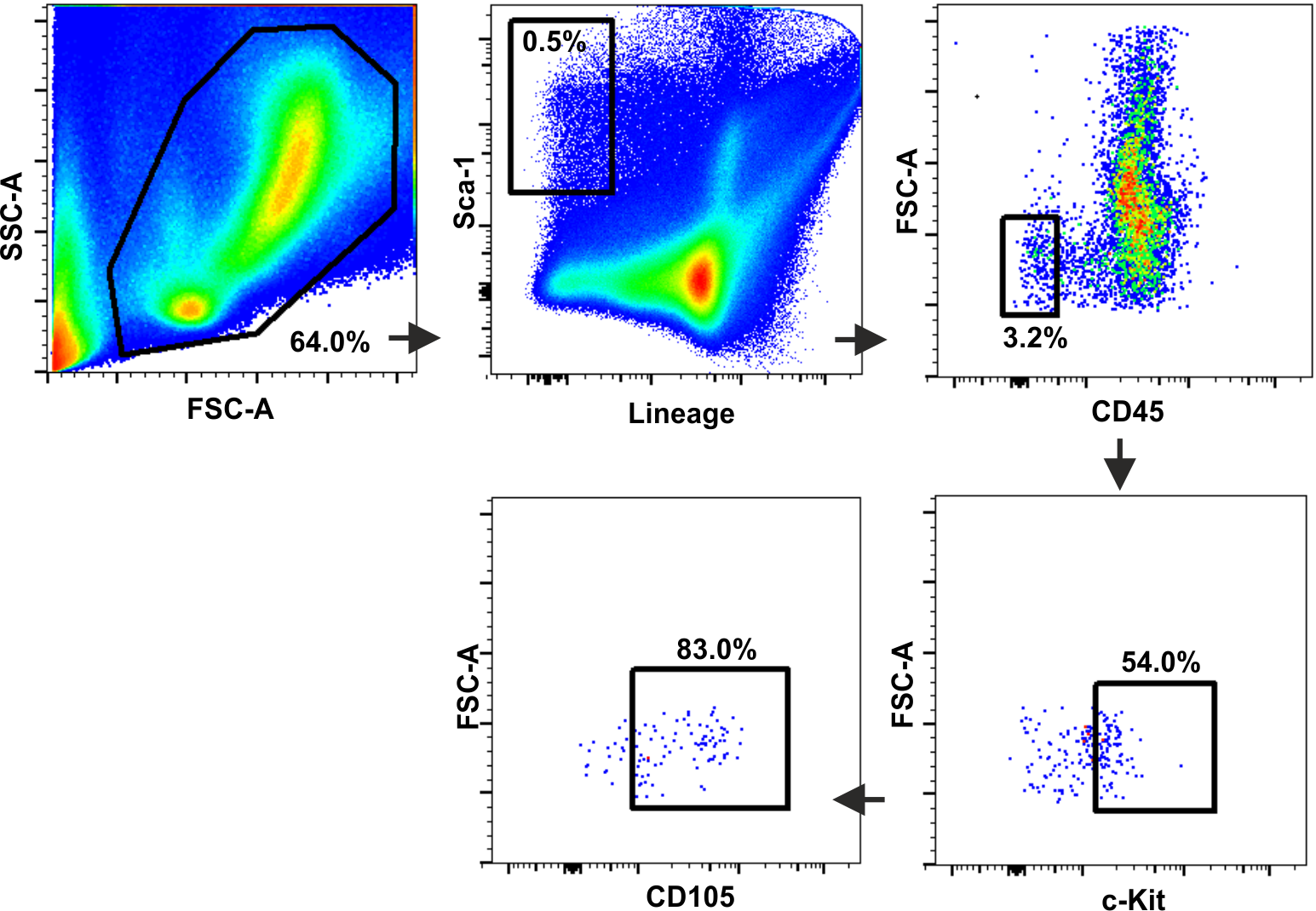

Supplement: Figure S5 — Flow cytometry analysis showing that SKL CD45−CD105+ subset significantly overlaps with Lin−Sca-1+CD45−c-Kit+FSClow subpopulation, with more than 70% of Lin−Sca-1+CD45−c-Kit+FSClow being CD105-positive. (TIF) [file pone.0063329.s005.tif]
